# Reappraisal of oxidized HMGB1 as a mediator and biomarker

**DOI:** 10.2144/fsoa-2022-0052

**Published:** 2023-02-10

**Authors:** Ross Pirnie, Kevin P Gillespie, Clementina Mesaros, Ian A Blair

**Affiliations:** 1 Center of Excellence in Environmental Toxicology & Department of Systems Pharmacology & Translational Therapeutics, University of Pennsylvania, Philadelphia, PA 19104, USA

**Keywords:** A-box. B-box, DAMP, DNA-binding, HMGB1, immune response, plasma biomarker, post-translational modification, RAGE, TLR

## Abstract

HMGB1 is a dual-function protein that acts as a chromatin-binding protein and as a danger-associated molecular pattern (DAMP) when released from activated immune cells or injured tissue. In much of the HMGB1 literature, immunomodulatory effects of extracellular HMGB1 are proposed to depend on its oxidation state. However, many of the foundational studies for this model have been retracted or flagged with expressions of concern. The literature on HMGB1 oxidation reveals a diversity of redox proteoforms of HMGB1 that are inconsistent with current models of redox modulation regulating HMGB1 secretion. A recent study of acetaminophen toxicity has identified previously unrecognized HMGB1 oxidized proteoforms. HMGB1 undergoes oxidative modifications that could serve as pathology-specific biomarkers and drug targets.

## Tweetable abstract

HMGB1 undergoes oxidative modifications that could serve as pathology-specific biomarkers and drug targets.

## Graphical abstract

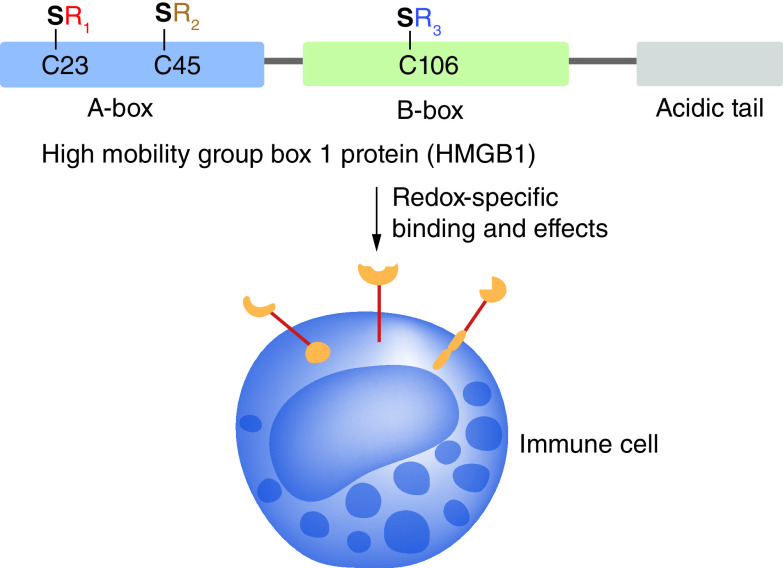

HMGB1, a non-histone 30 kDa chromosomal protein, was first isolated as a heparin binding protein (p30) with neurite outgrowth activity [1] and was originally called amphoterin [2]. The primary structure of HMGB1 (also known as HMG1) was deduced from its nucleotide sequence as a 215 amino acid single-chain polypeptide [3]. It contains A-box (9–79) and B-box (88–162) DNA-binding domains, an acidic C-terminal tail (186–215), and several redox-sensitive amino acids, including three highly conserved cysteine (Cys)-residues. (Figure 1). The N-terminal methionine-1 (M1) is lost during translation leaving glycine-2 (G2) at the amino terminus, although some reports refer to it as G1 and shift the labeling on all the other amino acids by -1.

**Figure 1.**
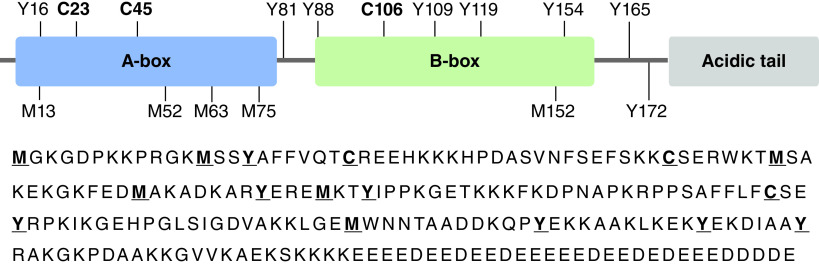
Amino acid sequence of HMGB1 with select redox-sensitive amino acids bolded and underlined (M, Y, C). A-box = amino acids 9–79, B-box = amino acids 88–162, acidic tail at C-terminus = amino acids 186–215 [4].

HMGB1 is a multi-functional protein that acts as a DNA chaperone as well as a damage-associated molecular pattern (DAMP) with immune-stimulating effects. HMGB1 localizes to the nucleus where it binds DNA, facilitates ATP-utilizing chromatin assembly factor/chromatin accessibility complex (ACF/CHRAC)‐dependent nucleosome sliding [5] and allows assembly of transcription factors [6,7]. The regulatory effects of HMGB1 on transcription are such that it is highly conserved across species and produced in all nucleated eukaryotic cells [8]. Extracellular HMGB1 can induce a range of immunological effects including inflammation [9], migration of maturing dendritic cells [10], and release of cytokines such as tumor necrosis factor α (TNFα) [11]. Neutralizing anti-HMGB1 antibodies significantly decrease the lethality of injuries from acetaminophen-overdose, demonstrating HMGB1's contribution to pathological inflammation [12].

Although the presence of HMGB1 in the nucleus is supported by its binding to DNA and chromatin protein partners, it has also been suggested that the intracellular localization of HMGB1 is regulated by post-translational modifications (PTMs). For example, it was reported that post-translational methylation of HMGB1 at lysine-43 (named as lysine-42 with N-terminal glycine-1) is responsible for its cytoplasmic localization in neutrophils [13]. HMGB1 was shown to be secreted from monocytes via a non‐classical, vesicle‐mediated secretory pathway [14]. Bonaldi *et al.* suggested that hyperacetylation of HMGB1 was required to redirect it toward secretion from monocytes [15]. In contrast, Youn and Shin found that phosphorylation of six serine residues in purported nuclear localization sequence (NLS) regions was required in order to control the nucleocytoplasmic shuttling of HMGB1 and redirect it toward secretion [16]. PTMs of HMGB1 may additionally modulate receptor binding of HMGB1 released extracellularly and therefore alter its extracellular function.

HMGB1 can be released in multiple pathological contexts and bind to a variety of extracellular receptors. Release of HMGB1 can occur passively from necrotic cells [9] or during regulated apoptosis of certain cell types [17]. Since nuclear HMGB1 is bound tightly to chromatin during apoptosis and secondary necrosis, the majority of HMGB1 released during apoptosis is likely to come from the cytoplasmic pool of HMGB1, which could vary in amount by cell type [17]. Active secretion of HMGB1 from activated cells is also possible, at least in part through an innate immune response mediated by both the cluster of differentiation (CD)14‐ and TNF [18]. The immunological effects exerted by extracellular HMGB1 (also known as amphoterin) are mediated through multiple receptors, including the receptor for advanced glycation end products (RAGE) (Figure 2) [19]. Secreted HMGB1 directly activates toll-like receptor (TLR) 2 and TLR4 [20]; whereas TLR9-dependent activation by DNA-containing immune complexes is mediated by HMGB1 and RAGE [21]. Therefore, HMGB1 binds to and activates multiple TLRs [22]. In addition, HMGB1 can recruit inflammatory cells to damaged tissues by forming a complex with chemokine ligand 12 (CXCL12) and binding to chemokine receptor 4 (CXCR4) (Figure 2) [23].

**Figure 2.**
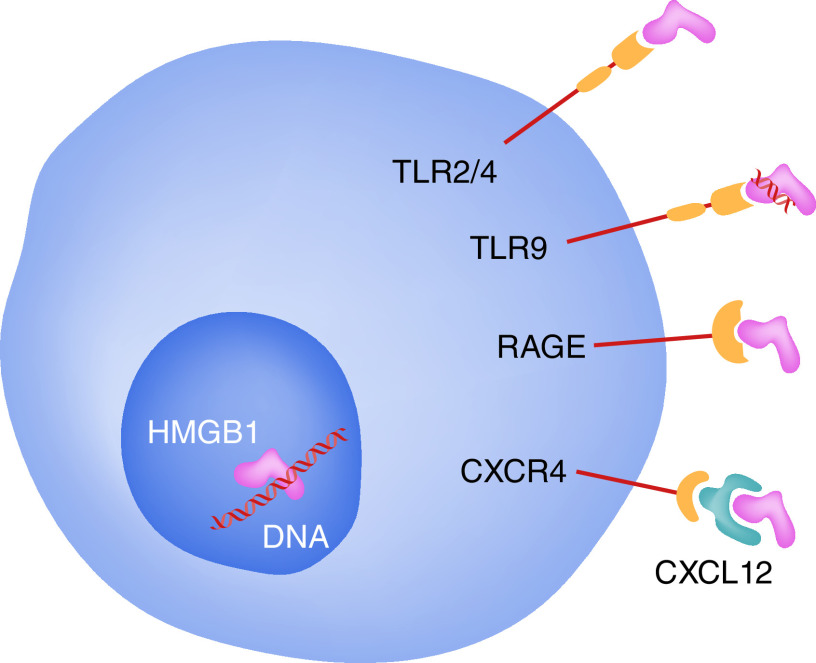
HMGB1 induces cytokine and chemokine effects through multiple binding partners RAGE, TLR2 and TLR4, via a complex with CXCL12/CXCR4, and TLR9 via a complex with RNA/DNA. Binding to the individual receptors might be redox sensitive [19–22].

Most studies of HMGB1 oxidation states and their effects on function either only broadly characterize HMGB1 as reduced and oxidized or allow for a limited number of oxidized Cys-residue proteoforms. Many studies additionally characterize HMGB1 oxidation state via gel mobility shifts or targeted mutation techniques that may not account for all proteoforms present. A priori limitation of the possible HMGB1 oxidized proteoforms simplifies experimental models but may not accurately reflect the complexity of HMGB1 oxidation and its effects on immune function *in vivo*.

HMGB1 release has been associated with oxidative stress in a wide range of pathological conditions. Reactive oxygen species (ROS) and reactive nitrogen species (RNS) can act both as redox signaling messengers and agents of oxidative damage depending on their concentration and a cell's antioxidant capacity. Many pathological conditions associated with extracellular HMGB1 release such as drug-induced hepatic necrosis, myocardial ischemia/reperfusion injury, and sepsis are also associated with excessive generation of ROS/RNS [24–29]. Originating from the mitochondria or other sources, ROS/RNS generated during pathological events may include hydrogen peroxide, superoxide and peroxynitrite as well as hydroxyl, nitrite, and carbonate radicals among others, each of which has its own reactivity and specificity [30]. Radical scavengers and antioxidants that would normally prevent the accumulation of ROS/RNS in cells are often depleted in conditions of pathological oxidative stress, increasing the likelihood of interaction between oxidants and intracellular proteins like HGMB1. Extracellular HMGB1 can therefore reasonably be expected to have exposure to a range of ROS/RNS species and concentrations depending on the context of release.

A range of unexplored oxidative modifications on Cys-, Met-, and other residues are possible and may account for the inconsistency in functional consequences of oxidation. There are five possible oxidation states of the three Cys-residues (Cys-disulfide [31], sulfenic acid (Cys-SOH) [32], sulfinic acid (Cys-SO_2_H) [33], Cys-SO_3_H [32,34], S-nitroso-Cys [35,36]) meaning that there are thousands of possible proteoforms without considering the additional complexity that could arise from oxidation of the Met-residues [37,38] or oxidation and nitration of the seven tyrosine residues (Figure 3) [39,40].

**Figure 3.**
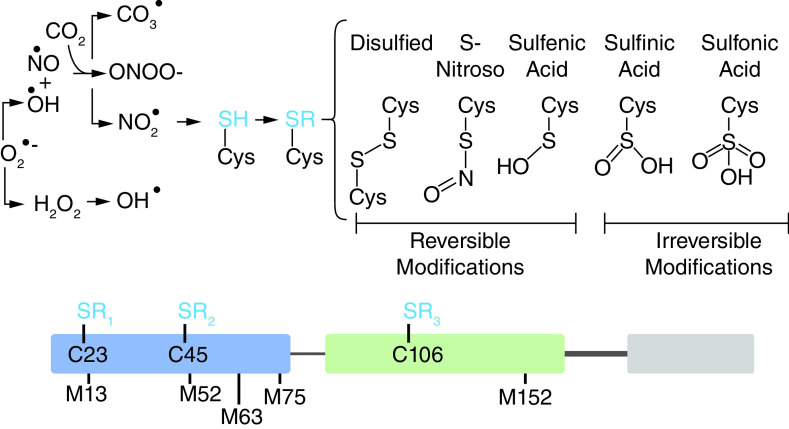
A great number (>1E3) of oxidized modification combinations are possible depending on the ROS/RNS species present and their respective concentrations. ROS and RNS like hydrogen peroxide (H_2_O_2_), peroxynitrite (ONOO-), superoxide (O_2_^•-^) and other radical oxidants can induce formation of reversible and irreversible Cys-residue modifications [31–36].

Oxidative modifications have distinct stability, reversibility and cellular lifetime, which can all inform on the biological context in which each would be expected to occur. Exposure of a Cys-residue to ROS results first in formation of a Cys-SOH, and although protein Cys-SOHs have been detected in cells [41], they are readily further oxidized to Cys-SO_2_s [42] or spontaneously form a disulfide bond with a free sulfhydryl group [43]. Like disulfides, Cys-SOHs are readily converted to thiols by reducing agents such as dithiothreitol (DTT), which must be considered during sample preparation and analysis of oxidized proteins. In contrast, Cys-SO_2_Hs are resistant to non-enzymatic reduction [44] but are readily further oxidized to Cys-SO_3_H. Cys-SNO is formed when nitric oxide (NO), peroxynitrite (ONOO-) or other S-nitrosylating agent reacts with a free sulfhydryl to form this readily reversible nitrosylated residue [45,46]. Met-residues are also readily oxidized upon ROS exposure to give Met-sulfoxide (Met-SO). Similar to Cys-SOH, Met-SO can be enzymatically reduced as well as further oxidized to Met-sulfone (Met-SO_2_) [47]. Variation in the concentration of ROS/RNS and duration of intracellular exposure in different pathological conditions creates potential for a multitude of different HMGB1 proteoforms with a mix of Cys-, Met- and other amino acid oxidation states.

Oxidative modifications, in a similar manner to all PTMs, may have unique structural and functional consequences that affect extracellular binding relationships and inform the immunological response to secreted HMGB1. Cys-oxidation under non-pathological conditions can allow Cys-residues to act as regulatory redox switches, responding to the local redox environment and modifying protein function accordingly. The NF-E2-related factor 2 (Nrf2)/ Kelch-like ECH-associated protein 1 (Keap1) system, for instance, allows release of Nrf2 into the nucleus in response to oxidative stress via oxidation of three Cys-residues in Keap1, which under reducing conditions confines Nrf2 to the cytosol ready for ubiquitination and degradation [48]. Under both pathological and non-pathological conditions, Cys-residue oxidation can have steric and hydrophobicity effects that alter protein structure and therefore receptor binding. Protein oxidation and the subsequent structural changes are often associated with loss of function, as was proposed in the incumbent HMGB1 oxidation state function paradigm. However, protein oxidation can sometimes induce a gain-of-function. For instance, oxidation of the plasma protein α2-macroglobulin increases its binding affinity for secreted cytokines, encouraging resolution of inflammation following release of oxidants from neutrophils [49]. Terminal oxidation of Cys-residues to Cys-SO_3_Hs has traditionally been viewed as exclusively inactivating in literature reports, but there is a possibility for future discovery of immune-activating effects, especially considering its association with pathological oxidation. Met-oxidation similarly has potential to alter protein structure and often occurs in parallel to Cys-oxidation; oxidation of α2-macroglobulin, for example, involves not only Cys-oxidation but also oxidation of Met-residues to Met-SO [49]. Met-SO has a “stiffer” and more polar side chain than an unmodified Met-residue with a similar hydrophobicity to lysine, a positively charged amino acid [37]. Oxidation of Met-residues has traditionally been associated with protein inactivation much like Cys-SO_3_H, but evidence is growing for alternative functions [37]. For instance, oxidation of Met-45 of inhibitor of kappa B-alpha (I*k*B*α*) increases its resistance to protein-degradation, thereby enhancing its ability to inhibit transcription factor nuclear factor kappa B NF*k*B) [50]. Therefore, additional studies are required to determine precisely how terminal Cys-oxidative modifications and Met-oxidations alter HMGB1 structure and to assess the subsequent changes to extracellular receptor binding affinity.

## Unreliable reports of HMGB1 & oxidized HMGB1 as biomarkers

Findings in many previous studies have encouraged interest in HMGB1's potential utility as a biomarker for a range of pathological conditions. Unfortunately, many of these studies were conducted using serum rather than plasma [51]. It was shown, using matched plasma and serum samples from twenty healthy control subjects, that HMGB1 is secreted when blood is allowed to clot to form serum [51]. Clotting resulted in a 30-fold increase in HMGB1 concentrations from 0.2 ng/ml in plasma to 6 ng/ml in serum. This would make it almost impossible to detect an increase in the release of HMGB1 from tissues into the plasma. Therefore, measurements in serum only reveal the capacity of blood cells to produce HMGB1 and do not reflect the circulating levels of HMGB1. Despite this fundamental problem, serum is still often used in biomarker studies, to quantify the HMGB1 that is secreted from tissues in different pathological conditions. Furthermore, much of the literature on HMGB1 and its oxidized forms as biomarkers is confused by retractions and temporary removal of papers [52–60] or expressions of concern about the data reported [31,61–70]. Studies that have monitored HMGB1 as a biomarker that must now be re-evaluated because of these issues include those on epilepsy [60], drug-induced liver injury [68], acetaminophen hepatotoxicity [61,65], asbestos exposure [67], and autophagy [69]. Studies that have specifically examined HMGB1 oxidation that require re-evaluation include, dietary regulation of acetaminophen toxicity [61], cytokine activity [63], macrophage activation syndrome [66], inflammasome activation [56], and neutrophil-mediated injury [65].

## Current paradigm for oxidized HMGB1 as a mediator & a biomarker

**Figure 4.**
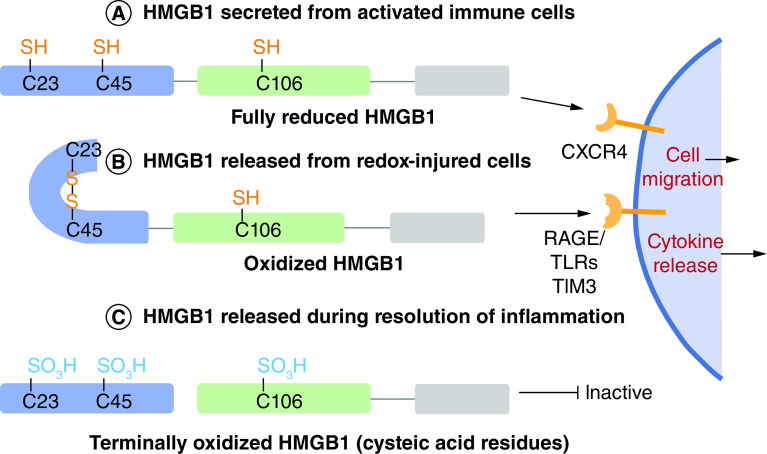
Currently accepted paradigm for the effect of Cys-oxidation on the activity of HMGB1. **(A)** Reduced HMGB1acts a chemokine through CXCR4. **(B)** HMGB1 with a thiol Cys-106 and a disulfide between Cys-23 and Cys-45 acts as a cytokine through RAGE, TRL's and TIM3. **(C)** Terminally oxidized HMGB1 is functionally inactive [71,72].

It has been widely proposed that the oxidation state of HMGB1 and its extracellular function are linked in a trinary relationship through its three Cys-residues - Cys-23, Cys-45, and Cys-106 (Figure 4). This model suggests that HMGB1 Cys- residues could be fully reduced, oxidized with a disulfide between Cys-23 and Cys-45 with Cys-106 reduced, or terminally oxidized at all three Cys-residues [71]. The three oxidation states were proposed to correspond to chemokine functionality, cytokine functionality, and inactivity, respectively. Sequential release of each oxidized HMGB1 proteoform from injured cells as pathological oxidative stress progressed would therefore correspond to each stage of inflammation - leukocyte recruitment, leukocyte activation, and eventual resolution of inflammation (reviewed in Lu *et al.*) [72].

Apart from retracted or flagged publications, the accepted paradigm for HMGB1 oxidation-function was supported by the finding that under mild oxidizing conditions, disulfide bond formation occurs between Cys-23 and Cys-45, but Cys-106 remains in the reduced form, and that this bond formation regulates the function of HMGB1 in response to oxidation [73]. Additional support for the accepted paradigm was derived from the finding that caspase-mediated oxidation of HMGB1 at Cys-106 induced immunological tolerance in apoptotic cells, whereas formation of the Cys-23/Cys-45 disulfide did not affect the activity of HMGB1 in this setting [74]. In contrast, binding of histone H1 to DNA was modulated by formation of the Cys-23/Cys-45 disulfide [75].

**Figure 5.**
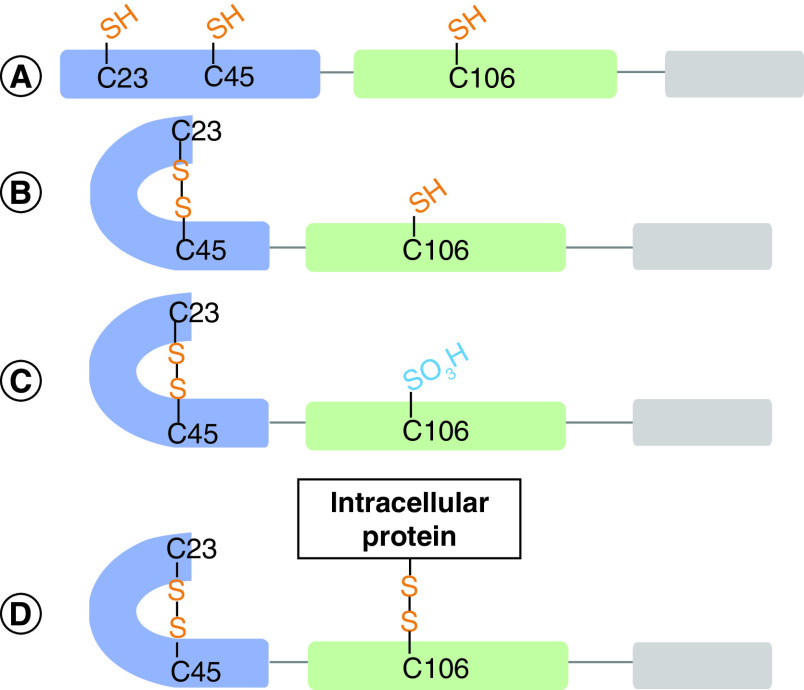
Oxidized HMGB1 proteoforms detected by liquid chromatography-tandem mass spectrometry (LC–MS/MS) following treatment of hepatocarcinoma cells with high concentrations of acetaminophen typically found in overdose patients.

However, oxidation of all three Cys-residues was found to improve TLR4 binding, cytokine release and immune responses [76]. In addition, oxidative stress elicited platelet/leukocyte inflammatory interactions through oxidative modifications to the three Cys-residues in HMGB1 [77]. Furthermore, LPS-induced kidney sepsis resulted in HMGB1 oxidation to the Cys-23/Cys-45 disulfide, which correlated with the ability of HMGB1 to induce inflammation [78]. Terminal oxidation of Cys-106 in HMGB1 to a cysteic acid (Cys-SO_3_H) was found to promote apoptosis and increase cell death upon exposure to chemotherapeutic agents [79]. It is noteworthy that mutation of Cys-106 to a serine residue inhibited its nuclear localization, ability to bind to TLR4, and inhibited cytokine release [80]. Yang *et al.* suggested that this provided evidence that Cys-oxidation to Cys-SO_3_H would have a similar effect [63]. However, there is an expression of concern related to this latter suggestion and no additional evidence has been obtained to date. Consequently, additional studies will be required to definitively establish how HMGB1 activity is modulated by such site-specific oxidations. Finally, analysis of HMGB1 proteoforms released in a cell-based model of APAP-overdose contradicted the currently accepted paradigm both in the types of oxidative modifications and their respective contexts of release [81]. Redox-injured cells released oxidized HMGB1 as well as fully reduced HMGB1, which was previously associated with release from activated immune cells (Figure 5A & B). Furthermore, two HMGB1 proteoforms not described in the previously accepted paradigm were detected: one with a mix of disulfide and terminally oxidized-Cys, the other with an intermolecular disulfide linkage at Cys-106 to an unidentified intracellular binding partner(s) (Figure 5C & D) [81]. Even without considering additional HMGB1 oxidized proteoforms, the proposed model of oxidation states and their relationship to function are clearly not as straightforward as suggested in numerous previous reports.

## Oxidized HMGB1 proteoforms as context-specific biomarkers

Regardless of the abundance of oxidative modifications and their functional consequences, certain modifications might have the potential to serve as pathology-specific biomarkers. Extracellular HMGB1 can originate from activated immune cell secretions in addition to other pathological sources. The presence and quantity of extracellular HMGB1 are therefore more indicative of general inflammation than specific pathological states. Oxidized proteoforms of HMGB1, however, may only arise in certain pathological conditions, thereby allowing for the potential identification of pathology-specific biomarkers. They have previously been proposed as potential biomarkers and drug targets by Venereau *et al.* [82] and others. However, future studies will need to avoid building on ideas emanating from the substantial number publications that have been retracted or temporarily removed [52–60] or statements of concern issued [31,61–70].

There are many pathological conditions associated with oxidative stress that result in the release of extracellular HMGB1 and which may be suitable for developing an oxidized HMGB1-based biomarker. Myocardial ischemia and reperfusion injury, particularly as a function of myocardial infarction, is associated with oxidative stress in the form of excessive hydrogen peroxide, superoxide and peroxynitrite generation [25]. Interestingly, peroxynitrite induces HMGB1 release by cardiac cells *in vitro* and HMGB1 upregulation in the infarcted myocardium *in vivo* [83]. In addition, oxidized HMGB1 was found to play a role in doxorubicin-induced peroxynitrite-dependent myocardial apoptosis [84]. Drug-induced liver injury (DILI) is the most common cause of fatal liver failure [85] and is often a consequence of reactive quinone metabolites that lead to excessive generation of ROS and RNS, as is the case in acetaminophen overdose, the most common cause of DILI [85–87]. Oxidized HMGB1 is secreted during acetaminophen-induced oxidant stress and cell injury in cultured mouse hepatocytes [88]. Similarly, oxidized HMGB1 mediates hepatic injury after murine liver ischemia-reperfusion [89]. HMGB1 release induced by liver ischemia involves TLR4-dependent ROS production and calcium-mediated signaling [90]. Finally, sterile inflammation is thought to be mediated by HMGB1 proteoforms that are released from acetaminophen-injured hepatocytes and this activation can be prevented by heparin analogs that bind to HMGB1 [12]. In most cases, the precise oxidation state of HMGB1 derived from pathological conditions has not been unambiguously characterized, opening up the possibility that unique proteoforms are among the oxidized fractions. Future work characterizing the oxidation state of HMGB1's Cys- and Met-residues as well as other residues of interest such as tyrosine, might yield specific, predictive biomarkers.

The lack of unambiguous HMGB1 oxidized proteoform characterization in different pathological conditions can, at least in part, be attributed to the challenging analytical requirements. Specific antibodies for each Cys-oxidation state are not available and although anti-Met-SO antibodies have been produced and sold commercially, subsequent studies have shown that these antibodies are not specific [91]. Reactive, reversible modifications sensitive to the redox environment pose a challenge for sample preparation, requiring care that endogenous modifications are not lost, while ensuring that artifactual modifications are not created. Despite these challenges, technologies sufficient for robust characterization of oxidized proteoforms exist and may aid in the development of specific biomarkers (reviewed in [92]). Derivatization and subsequent analysis by western blotting, LC–MS/MS or other methods can provide oxidation state information. Readily reversible Cys-oxidation states such as disulfide linkages can be differentiated from free thiols with derivatization, followed by reduction and additional derivatization, which can then be analyzed by quantitative LC–MS/MS [81]. In addition, Cys-SOH, a reversible oxidative modification, can be detected using dimedone-based chemical probes [93]. Dimedone reacts selectively with the sulfur atom in Cys-SOH to form a stable thioether linkage that can be attached to biotin reporter tags and detected directly by LC–MS/MS [94]. Cys-SNO can similarly be detected using derivatization approaches such as the biotin switch technique, wherein free thiols are first blocked by a thiol-specific methanethiolation reagent such as methyl methanesulfonate, followed by ascorbate-mediated conversion of Cys-SNO groups back to Cys-residues and S-biotinylation of the Cys-free thiols [95]. Purification of the biotinylated proteins with streptavidin columns followed by western blotting with specific antibodies facilitates characterization of the proteins that originally had Cys-SNO residues [96]. Specific sites of Cys-CNO cysteine residues can be characterized by replacing the biotinylation step with derivatization using an isotope coded affinity tag (ICAT), followed by trypsin digestion, and LC–MS/MS analysis of the resulting tryptic peptides [97]. Modifications resistant to non-enzymatic reversal such as Cys-SO_2_H, Cys-SO_3_H, Met-SO, and Met-SO_2_ can be detected directly by LC–MS/MS [81].

## Conclusion

The paradigm for the extracellular function and oxidation state relationship of HMGB1 lacks sufficient supporting data in light of numerous retractions, temporary removal of papers, and expressions of concern [31,52–70]. The precise oxidized HMGB1 proteoforms present in a variety of pathological and non-pathological conditions is unclear as many studies have failed to account for all of the potential proteoforms or limited their analysis to a binary “reduced” and “oxidized”. The previously accepted paradigm for oxidative structural changes in HMGB1 (Figure 4) may be far too simple and overlook many previously unexplored proteoforms (Figure 3)

## Future perspective

It is now possible to characterize oxidized HMGB1 proteoforms with high specificity and sensitivity by the use of immunoaffinity purification coupled with efficient derivatization and stable isotope dilution LC–MS/MS [81]. This should permit the quantification of HMGB1 and novel oxidized proteoforms in plasma form different pathological conditions. Plasma should be used instead of serum because the preparation of serum causes a 30-fold increase in HMGB1 levels that would prevent the detection of any HMGB1 release from tissues into the plasma [51]. Potential pathological conditions where HMGB1 could be involved include: alcoholic liver disease [98], asbestos exposure and mesothelioma [67], pleural mesothelioma [99], acetaminophen hepatotoxicity [62], senescence [76], systemic lupus erythematosus [100], sepsis [101], type 1 and Type 2 diabetes mellitus [102], breast cancer [103], pancreatic cancer [104], squamous cell carcinoma [105], non-small-cell lung cancer [106], Alzheimer's disease [107], epilepsy [60], Parkinson's disease [108], atrial fibrillation [109], coronary artery disease [110], heart failure [111], and coronary artery stenosis [112]. Therefore, tools are now available to probe the full range of potential oxidized HMGB1 proteoforms, which should ultimately lead to the validation of useful specific biomarkers for different diseases as well as potential therapeutic targets [82] in the relevant diseases.

**Executive summary**

- HMGB1 is a dual-function protein that acts as a DNA-binding protein in the nucleus as well as a DAMP when released from activated immune cells or injured tissue.

**Unreliable reports of HMGB1 & oxidized HMGB1 as biomarkers**

- The paradigm for the extracellular function and oxidation state relationship of HMGB1 lacks sufficient supporting data in light of numerous retractions, temporary removal of papers, and expressions of concern.
- HMGB1 is secreted when blood is allowed to clot to make serum. Many studies have quantified HMGB1 in serum rather than plasma, so they do not reflect circulating levels but just the capacity for blood cells to secrete HMGB1.
- The precise oxidative HMGB1 proteoforms present in a variety of pathological and non-pathological conditions is unclear.

**Current paradigm for oxidized HMGB1 as a mediator & a biomarker**

- Many studies have failed to account for all the potential proteoforms or limited their analysis to a binary “reduced” and “oxidized” proteoform analysis.
- In view of the large number of potential oxidative HMGB1 proteoforms, there is ample opportunity for discovery of many specific oxidized proteoforms that might serve as biomarkers for specific pathological states.
- Unique oxidative modifications may additionally have implications for the structure and function of HMGB1 in the extracellular space as individual oxidized proteoforms could have different activities on receptors such as TLRs that mediate cytokine release.

**Oxidized HMGB1 proteoforms as context-specific biomarkers**

- Activation of sterile inflammation, which is thought to be mediated by HMGB1 released from acetaminophen-injured hepatocytes, can be prevented by heparin analogs that bind to HMGB1 and by anti-HMGB1 antibodies.
- A recent study of acetaminophen-mediated HMGB1 release from hepatocytes has identified previously unrecognized oxidized HMGB1 proteoforms.

**Conclusion**

- There is ample opportunity for the discovery of numerous context-specific proteoforms that might serve as biomarkers for specific pathological states.
- Unique oxidative modifications have implications for the structure and function of HMGB1 in the extracellular space.
- The previously accepted paradigm for oxidative structural changes and HMGB1 may be far too simple and overlook many previously unexplored proteoforms.

**Future directions**

- The development of highly specific and sensitive methodology based on the use of immunoaffinity purification coupled with efficient derivatization and stable isotope dilution LC–MS/MS should permit the characterization and quantification of novel oxidized HMGB1 proteoforms in plasma (not serum) obtained from different pathological conditions.
- Tools are now available to probe the full range of potential oxidized HMGB1 proteoforms, which should ultimately lead to the validation of useful specific biomarkers for different diseases as well as potential therapeutic targets in those diseases.
